# Clinical Outcomes of Single Tooth Implant Placement in the Mandibular Region: A Case Report

**DOI:** 10.7759/cureus.66379

**Published:** 2024-08-07

**Authors:** Bhawana J Dajjuka, Seema R Kambala, Mithilesh M Dhamande, Dhanashree A Minase, Pragati Agrawal

**Affiliations:** 1 Dentistry, Sharad Pawar Dental College and Hospital, Datta Meghe Institute of Higher Education and Research, Wardha, IND; 2 Prosthodontics and Crown and Bridge, Sharad Pawar Dental College and Hospital, Datta Meghe Institute of Higher Education and Research, Wardha, IND

**Keywords:** missing tooth, osseointegration, prosthesis, abutment, dental implants

## Abstract

The most frequent reasons why people lose their teeth are trauma, dental caries, developmental defects, and genetic abnormalities. Dental implants have become a popular alternative to traditional dentures and bridges due to their ability to restore function through osseointegration. This case report presents a 35-year-old male with a two-year history of a missing lower right molar due to caries. With no systemic health issues or harmful habits, the patient underwent dental implant placement after a thorough evaluation and cone-beam computed tomography (CBCT) analysis. A parallel-sided, threaded implant was placed in the 46 region. Postoperative care included antibiotics, analgesics, and follow-up visits. Second-stage surgery was done, which was followed by impression-making and healing abutment placement. The final crown was cemented with careful occlusion verification. The patient exhibited excellent healing and was scheduled for regular follow-ups to ensure successful implant integration and function restoration.

## Introduction

Dental caries, trauma, developmental defects, and genetic disorders are the most common causes of tooth loss. Over the last three decades, dental implants have become more popular among people who have lost their teeth and want to have them restored [1,2]. Dentures and bridges were the options available for replacing the missing teeth before dental implants came into the picture. A removable denture, principally consisting of porcelain or acrylic resin, is inserted in a plastic base and retained by the hard and soft tissue of the mouth. According to the presence or absence of teeth in an arch, a denture can be classified as partial or complete. A fixed partial denture (FPD) is indicated when one or more teeth are missing in a dental arch. A bridge is made out of one or more artificial teeth made of metal, porcelain, or gold [3]. With advancements in dental implant technology, there is now a desirable substitute for traditional dentures and bridges. Osseointegration, in which osteoblasts develop and immediately integrate with the surface of titanium posts surgically placed into the jaw, is the fundamental process underlying dental implants [4,5]. Dental implants, which have gained immense popularity in the past few decades, allow patients who are partially or totally edentulous to regain function close to normal.

## Case presentation

A 35-year-old male patient reported to the Department of Prosthodontics and Crown and Bridge with a complaint of a missing tooth in the lower right back region of the jaw for two years. The medical history showed no systemic health issues, harmful habits, or known allergies. The dental history showed that tooth 46 was extracted two years ago due to caries. The general evaluation of the patient's periodontal health was satisfactory. After considering the advantages and disadvantages of the many treatment choices that were given to the patient, it was decided to replace the missing tooth with an implant.

**Figure 1:**
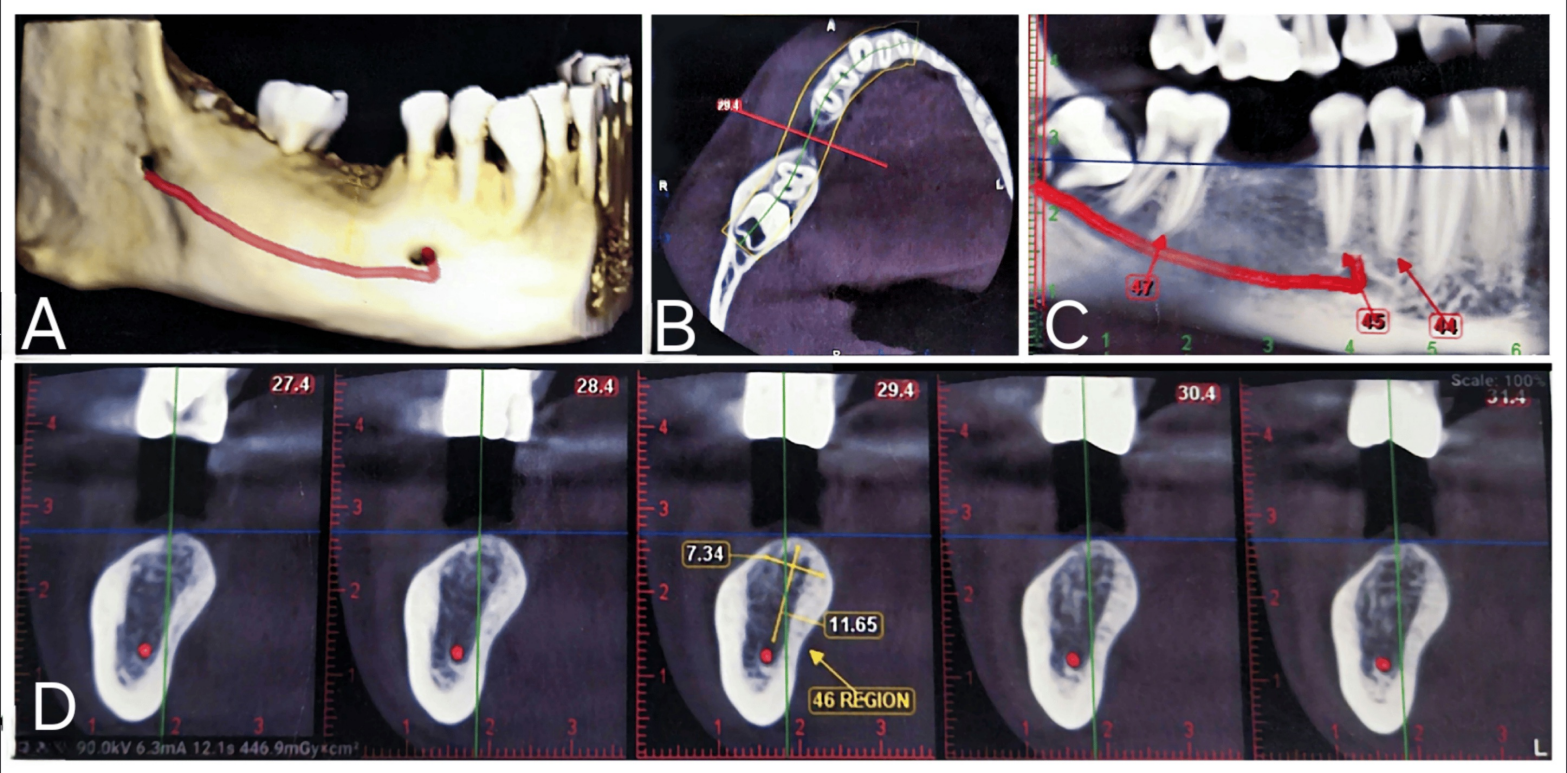
Detailed CBCT images of the mandibular region (A) Three-dimensional view of the mandible; (B) axial section of the mandible in the missing tooth region; (C) sagittal section of the mandible in the missing tooth region; (D) cut section of the missing tooth in the sagittal section, showing the dimensions of the bone CBCT: cone-beam computed tomography

Diagnostic examinations, such as cone-beam computed tomography (CBCT), and blood investigations were performed. According to the CBCT analysis, bone width is 8 mm and bone type is D2 (porous cortical and coarse trabecular according to the Misch classification). This led us to decide on the treatment plan for implant placement in the 46 region. Detailed CBCT images of the mandibular region are shown in Figure 1.

**Figure 2:**
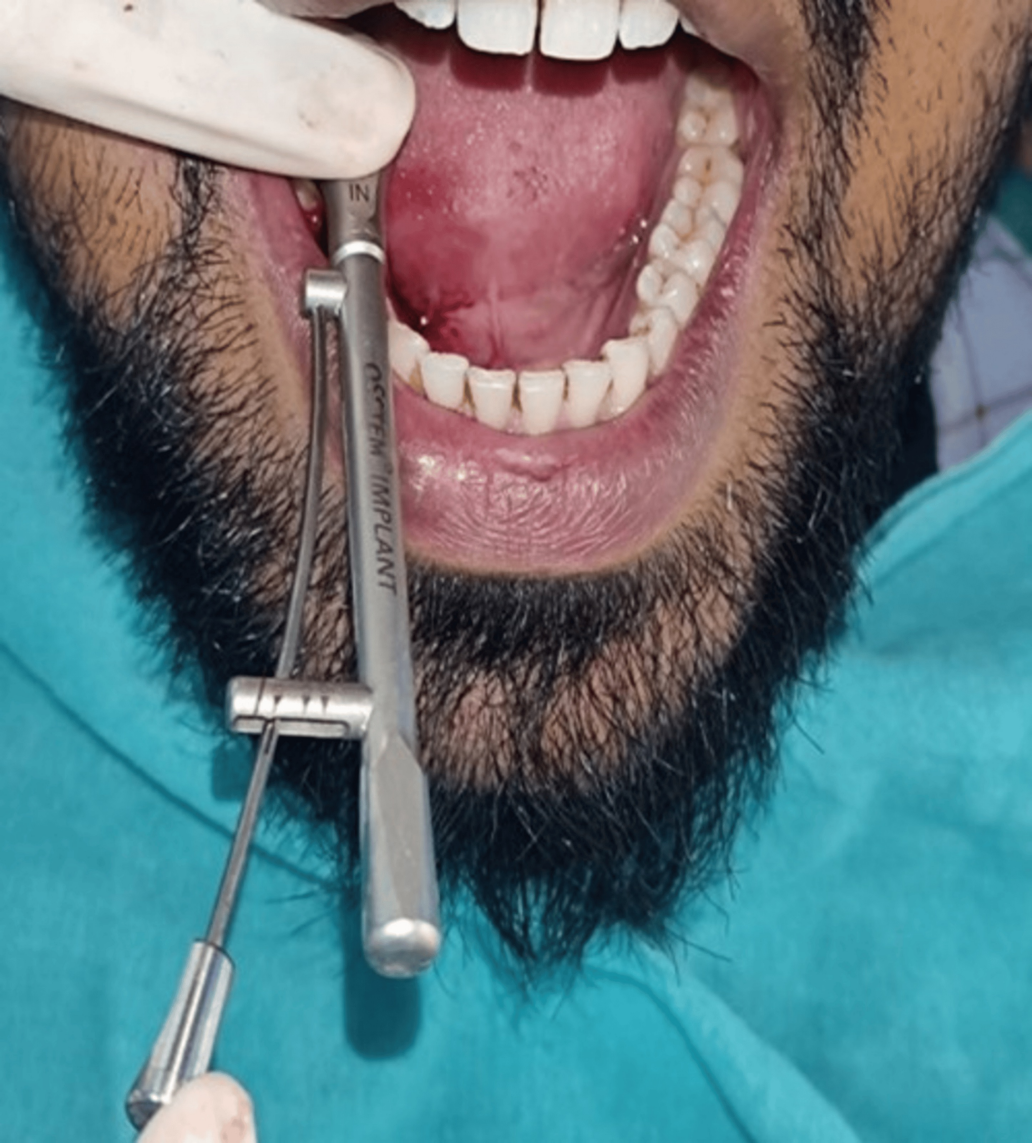
Image showing the primary stability measurement with a torque wrench

The surgical step includes an ostectomy using a lance drill following the manufacturer's instructions. A parallel-sided, threaded implant (OSSTEM Implant, Seoul, South Korea) with an internal hex type of connection was then placed. The implant dimensions were 5.0 mm in diameter and 8.5 mm in height. Primary stability was measured with the help of a 35 N cm torque wrench, which was above the minimum required of 25 N cm for success (Figure 2).

**Figure 3:**
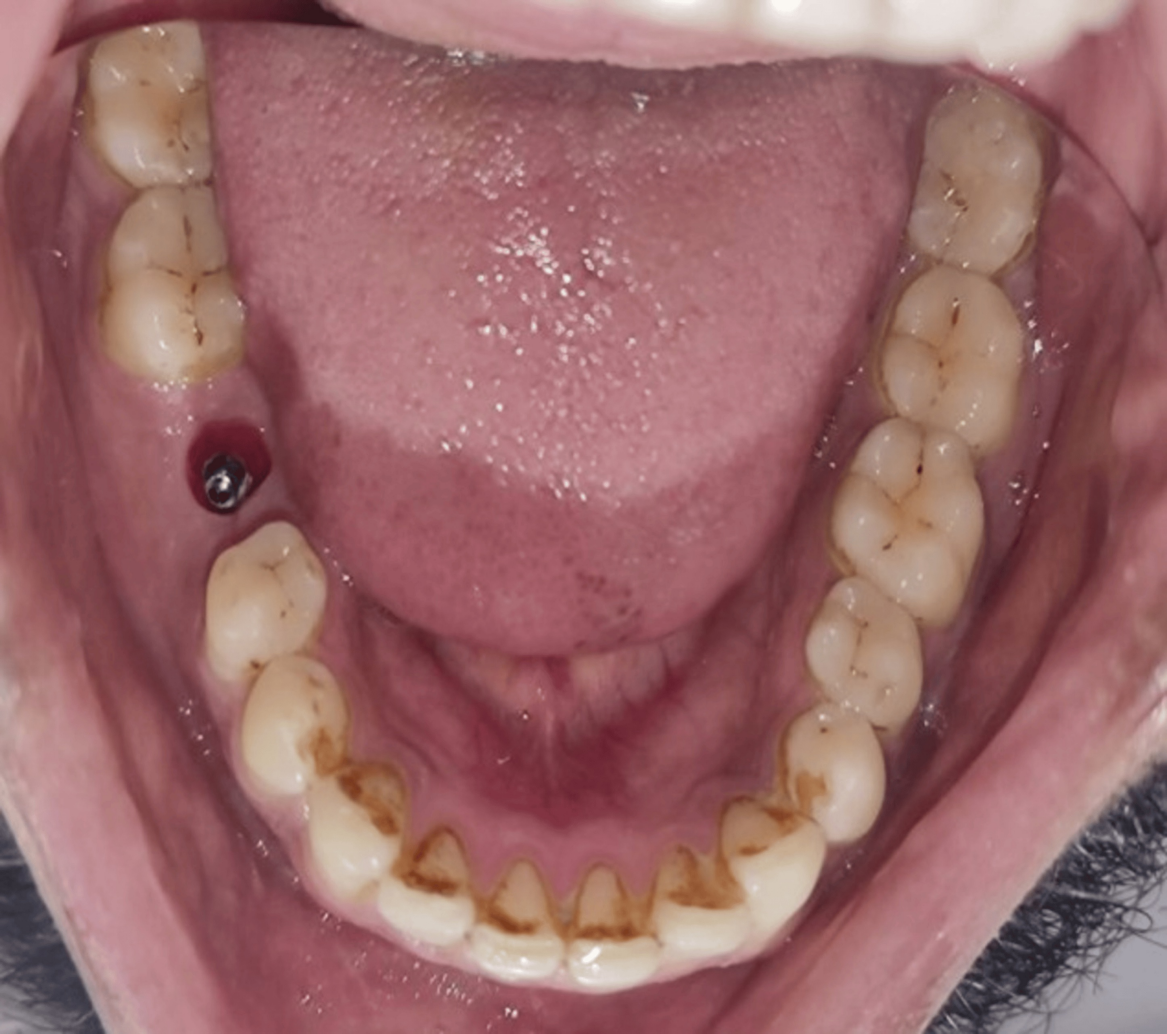
Image of the gingival collar post-second stage surgery

A cover screw was placed on top of the implant, and the flap was sutured back using silk 3-0. In addition to postoperative instructions, appropriate analgesics and antibiotics were prescribed. One week after surgery, the patient returned for a follow-up appointment to have his sutures removed with no signs of infection. Finally, three months after the implant placement, the second stage of surgery was performed, which included the removal of the cover screw and the placing of the gingival former (5 mm, regular). The evaluation of the implant was done with the help of an intraoral radiograph. When the patient was examined again after 15 days, the gingiva was found to be healing, and buccal contours exhibited a resemblance to those of the adjacent tooth, providing a natural emergence profile for the prosthesis (Figure 3).

**Figure 4:**
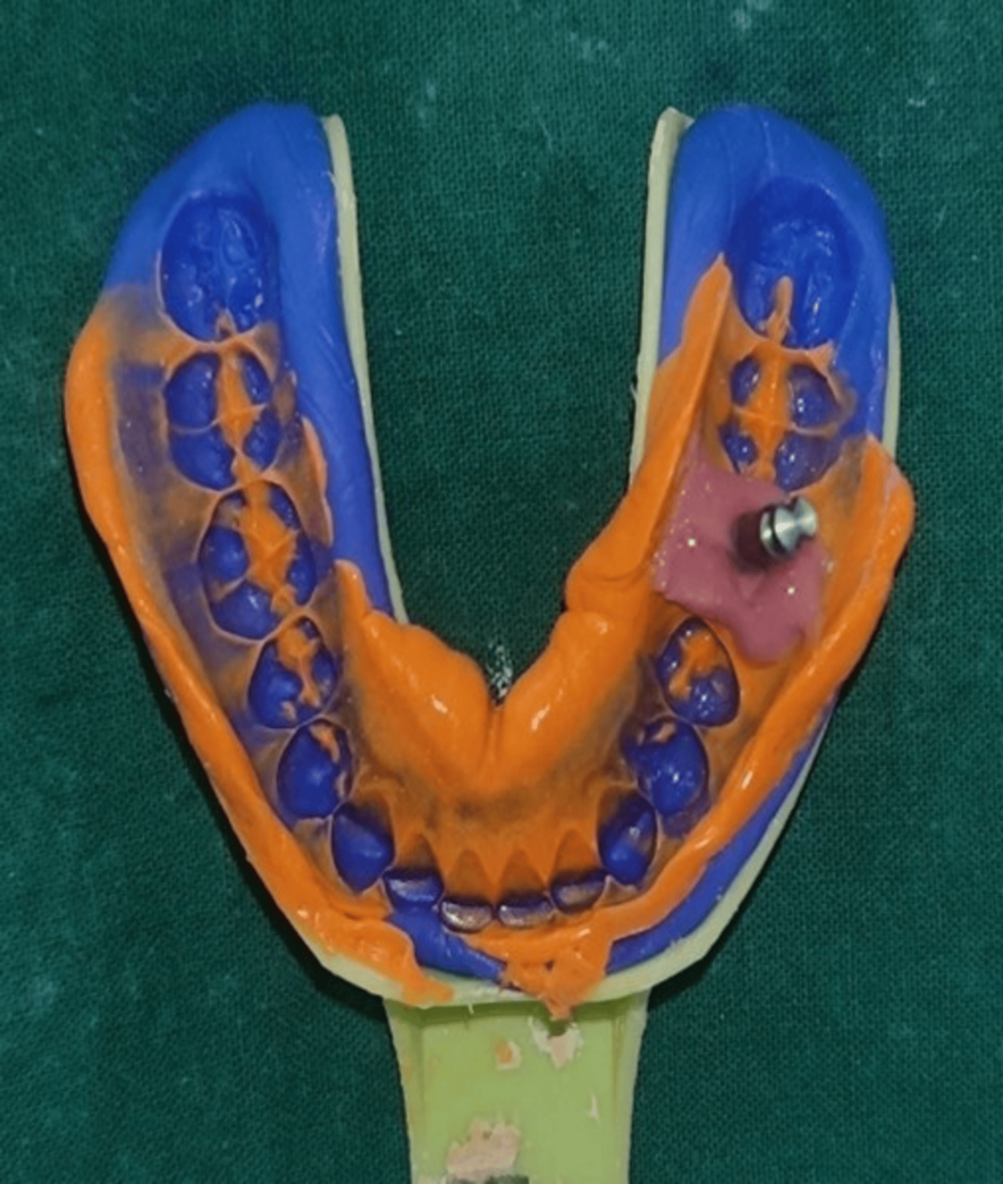
Elastomeric impression of the mandibular arch with transfer coping

An impression coping was inserted, followed by a closed-tray impression made using polyvinyl siloxane elastomeric material (AQUASIL, Dentsply/Caulk, Milford, Delaware) in putty and light body consistency to record the position of the implant. The two-step impression technique was used, where the first impression was made with the putty elastomer using a cellophane sheet in between. Subsequently, a second wash impression was made using the light body elastomeric impression material. Finally, the cellophane sheet was removed. Figure 4 shows an elastomeric impression of the mandibular arch in which a lab analog is attached in the mandibular right molar region and an esthetic mask was applied to mimic the gingiva in the stone model.

**Figure 5:**
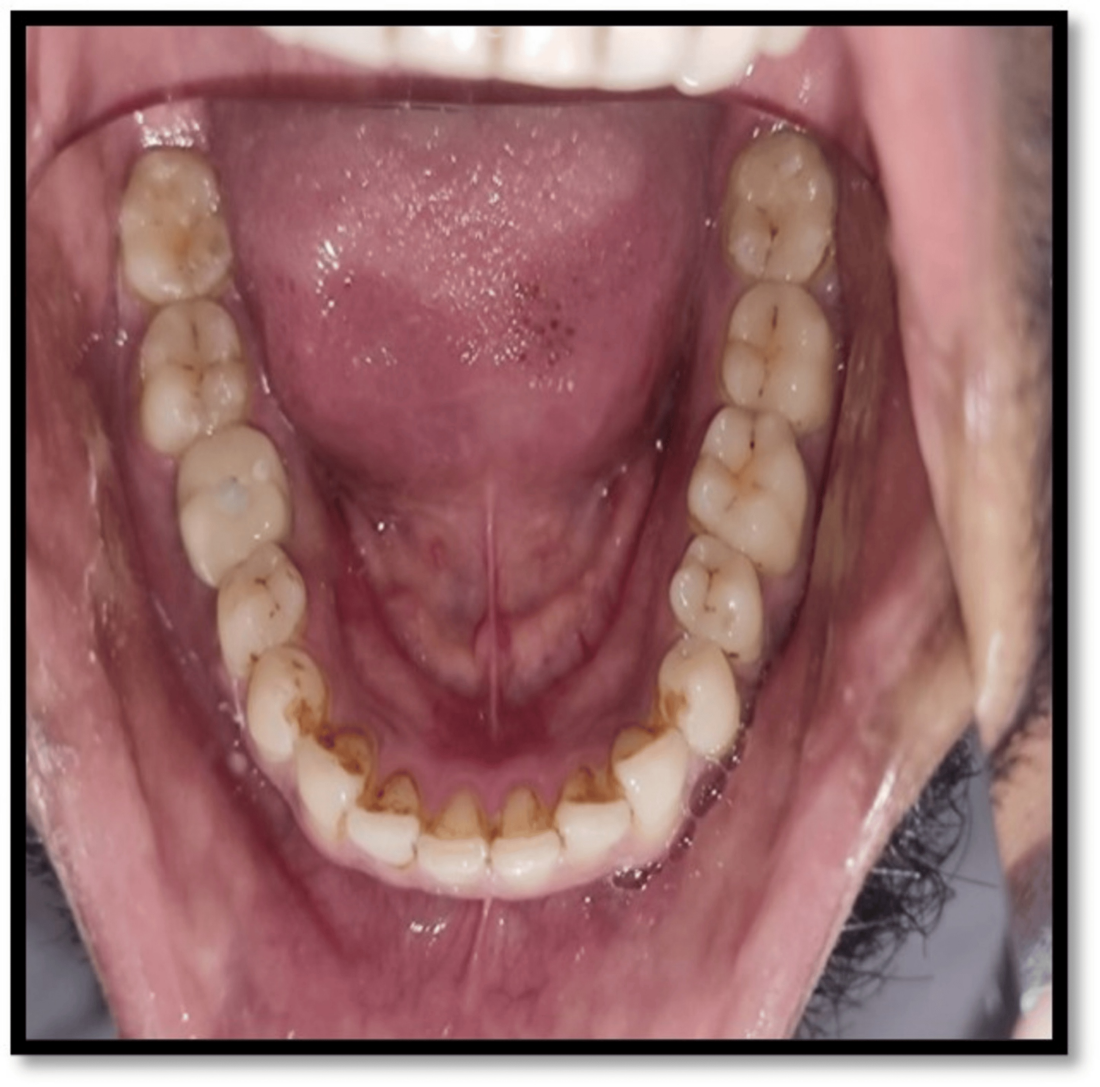
Post-cementation image of the porcelain-fused-to-metal crown prosthesis

The abutment height was selected according to the restorative space available, which was 4 mm. The abutment edges are to be positioned at least 0.5 mm below the gingival crest. The shade was recorded under natural sunlight at eye level using the classic Vita shade guide. After one week, the patient was recalled for follow-up. During this visit, the gingival former was removed, and the transfer abutment was positioned; an intraoral periapical radiograph was obtained to verify the abutment placement. With the use of a torque wrench, the abutment was then torqued to 25 N. The final crown prosthesis was cemented with glass ionomer cement (SHOFU Hy-Bond, Kyoto, Japan), and the contacts were checked in centric and eccentric movements. Figure 5 displays the post-cementation image of the porcelain-fused-to-metal crown prosthesis.

The patient was provided with detailed instructions regarding postoperative care, and the patient had follow-up appointments set after one, three, and six months. The subsequent follow-ups revealed a noticeable improvement in chewing function and esthetics. There were no postoperative complications or adverse events reported.

## Discussion

The basic principles of prosthetic rehabilitation, implant surgery, and treatment planning were all discussed in this case report. Dental implants are another viable treatment option for the management of missing teeth in the mandibular region. Fixed dental implant-supported prosthetics (FPD) have many advantages over conventional crowns and bridges or RPDs (removable partial dentures) [6]. Using dental implants offers numerous benefits, including the maintenance of residual bone, increased longevity, non-involvement of adjacent teeth, and ease of oral hygiene.

A precise surgical plan and preoperative preparation are required for a successful implant procedure to replace lost mandibular teeth. Technical expertise is also essential [7,8]. When making treatment plans, it is critical to take the finished prosthetic into account. A dental implant may not yield the desired outcome if the positioning of the prosthesis is not visualized preoperatively [9,10].

Resin-bonded bridges, FPDs, and RPDs were the alternative treatment choices for our strategy. While they are a viable alternative, RPDs have the potential to cause alveolar bone loss in the abutment and non-abutment teeth [11]. Furthermore, the percentage of people who are dissatisfied with RPDs ranges from 9% to 26% [12].

Conversely, the utilization of FPD would require the unnecessary destruction of adjacent teeth to prepare them for the role of abutments, which would lead to the loss of perfectly healthy tooth structure. An alternative would be a resin-bonded bridge, which has a significant risk of pontic failure and debonding but would lessen the damage to the adjacent teeth [13]. Therefore, the implant can be considered a better option. The clinician should consider the time period required for implant integration and soft tissue healing, the development of the emergence profile, the occlusal forces during progressive loading, and the occlusal forces acting on the final restoration. Dental implants provide several advantages, including ease of oral care, preservation of residual bone, non-involvement of adjacent teeth, and greater longevity [14,15].

## Conclusions

This case report demonstrates the successful use of a dental implant to replace a missing lower right molar in a 35-year-old male patient. The implant procedure was carefully planned and executed, leading to excellent primary stability and successful osseointegration. The patient reported significant improvement in chewing function and esthetics, with no postoperative complications observed. The following key outcomes were noted: (1) primary stability: the implant achieved a primary stability of 35 N cm, well above the minimum requirement for success; (2) healing process: the patient exhibited excellent healing at each follow-up visit, with a well-formed gingival collar and natural emergence profile; and (3) functional and esthetic outcomes: the final crown prosthesis provided satisfactory occlusion and improved the patient's overall oral function and appearance. This case highlights the following advantages of dental implants over traditional dentures and bridges: (1) the preservation of residual bone: the implant helped maintain the bone width of 8 mm, as confirmed by CBCT analysis; (2) non-involvement of the adjacent teeth: the treatment avoided unnecessary damage to the adjacent healthy teeth, preserving their integrity; and (3) ease of oral hygiene: the implant-supported prosthesis facilitated easier oral hygiene maintenance compared to removable dentures.

Regular follow-up appointments were crucial in ensuring the sustained success of the implant and optimal function restoration. The patient's positive response underscores the importance of thorough preoperative planning, precise surgical execution, and diligent postoperative care in achieving successful dental implant outcomes.
